# Antibody Titers After a Third and Fourth SARS-CoV-2 Vaccine Dose in Bizen City, Japan

**DOI:** 10.2188/jea.JE20230034

**Published:** 2023-09-05

**Authors:** Tomoka Kadowaki, Ayako Sasaki, Naomi Matsumoto, Toshiharu Mitsuhashi, Soshi Takao, Takashi Yorifuji

**Affiliations:** 1 Department of Epidemiology, Graduate School of Medicine, Dentistry and Pharmaceutical Sciences, Okayama University, Okayama, Japan; 2 Center for Innovative Clinical Medicine, Okayama University Hospital, Okayama, Japan

Antibodies against SARS-CoV-2 that are acquired through vaccination tend to decline over time. Previous studies examining the dynamics of immune responses after a second dose of vaccine for novel coronavirus disease 2019 (COVID-19) have demonstrated that elderly people had lower peaks and larger declines in antibody titers compared with younger people.^1^^,^^2^ Recent reports have also demonstrated that among health care workers, trends in antibody titers after a third and fourth dose were similar, and the declines were slower compared with that after a second dose.^3^^–^^5^ More information is needed regarding the dynamics of immune responses after these boosters, especially in vulnerable populations. Therefore, we examined the changes in antibody titers after the third and fourth doses of vaccination in the general population, including elderly people.

The Bizen COVID-19 Antibody Test project is a community-based survey that began on June 3, 2022, in which antibody titers are measured every 2 months among residents of Bizen City, Okayama Prefecture, located in the western part of Japan. The study was approved by the Ethics Committee of Okayama University Hospital (No. 2205-061), and all the participants provided written informed consent. We recruited 1,956 participants aged 18 years or older directly from among local residents or through local institutions, such as nursing homes, city hall, and other institutions in the city. In this analysis, we included up to the third measurement of antibody titers from participants who received at least a third vaccine dose and had no history of COVID-19 infection, as confirmed in a self-report questionnaire. This yielded a total of 2,868 measurements (937 from the first; 966 from the second, and 965 from the third measurement of antibody titers) from 1,862 participants. Among them, multiple measurements (ie, more than two measurements) were available for 1,720 participants (92.4%).

We collected fingertip whole blood samples (30 µL) and measured antibody titers targeting the spike protein receptor-binding domain using the Mokobio SARS-CoV-2 IgM & IgG Quantum Dot immunoassay (Mokobio Biotechnology R&D Center Inc., Rockville, MD, USA). Previous studies already demonstrated the validity of this measurement based on a high correlation between antibody titers measured with this device and those measured using serum samples.^6^^–^^8^ We also collected the vaccination status, including the type and the date of vaccination, of the participants from the Bizen City registry or the self-reported questionnaire. We then plotted the association between the number of days elapsed since the third or fourth vaccine dose and the antibody titer for each measurement using box-and-whisker diagrams. We also compared the levels of antibody titers among participants who had received their fourth vaccine dose, by age group (<65 and ≥65 years) using locally weighted regression smoothing. All analyses were conducted with Stata SE version 17 (StataCorp LP, College Station, TX, USA).

**Figure 1.**
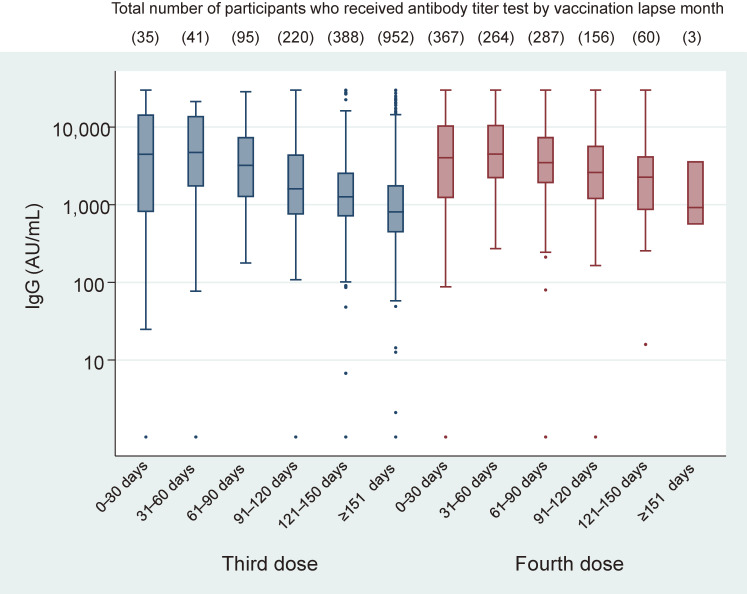
Immunoglobulin G (IgG) values after third and fourth SARS-CoV-2 vaccine doses. The upper limit of the test result was set at 30,000 AU/mL, according to the manufacturer’s instructions; any value exceeding this limit was replaced by the upper limit. There was a statistically significant difference between antibody titers 150 days after the third vaccination and 30 days after the fourth vaccination (*P* < 0.001), while there was no significant difference between peak antibody titers after the third and fourth vaccination (*P* = 0.85). *P*-values were calculated using the Mann-Whitney U test.

**Figure 2.**
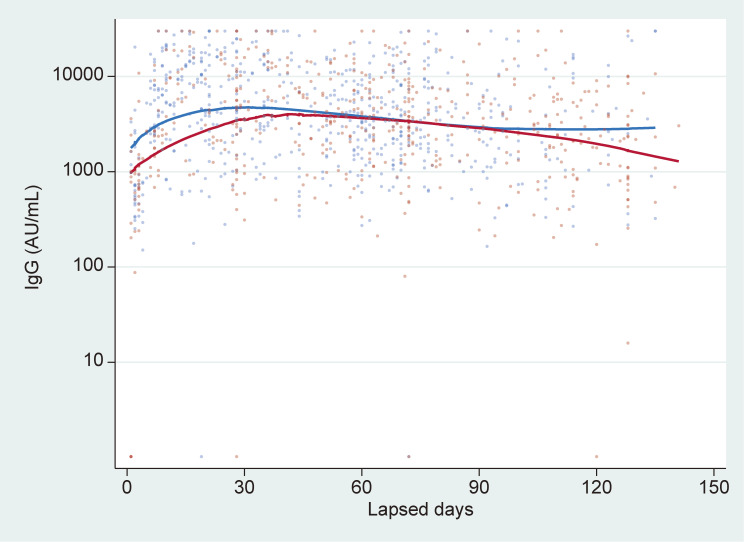
Immunoglobulin G (IgG) values after fourth SARS-CoV-2 vaccine dose, by age group. Individual antibody titers were overlaid by locally weighted scatterplot smoothing. Blue and red lines indicate age <65 and ≥65 years, respectively. The upper limit of the test result was set at 30,000 AU/mL, according to the manufacturer’s instructions; any value exceeding this limit was replaced by the upper limit. There was no statistically significant difference of antibody titers from 31 and 60 days after vaccination between the age groups (*P* = 0.70, calculated using the Mann-Whitney U test).

Approximately 65.8% of participants were women, with 1,345 and 517 participants in the age groups 18–64 and ≥65 years, respectively. The mean age of the participants who received the third dose of vaccination was 46.9 years, while that of those who received the fourth dose of vaccination was 60.7 years. Antibody titers approached the peaks at approximately 1 month after vaccination and gradually declined over time, with similar trends for the third and fourth vaccinations. Although the number of samples was small, the median antibody titer more than 151 days after the fourth vaccine dose was nearly the same as the median value the same number of days after the third vaccine dose (Figure 1). Antibody titers increased slower but declined earlier among older people compared with younger participants (Figure 2).

The present findings indicate that the changes in the immune responses decreasing over time after vaccination were similar between the third and fourth vaccinations, which was consistent with previous findings obtained among health care workers.^3^^–^^5^ Also, as shown in Figure 1, the changes in the immune responses may indicate waning vaccine effectiveness of the fourth vaccination and no substantial additional effectiveness over the third vaccination after more than 5 months have passed. Additionally, the elderly participants had a shorter durability of immune responses compared to younger participants. There are several limitations in the present study. We could not take into account the type of vaccination, the comorbidities of the participants, and the age category when we examined IgG values after the third and fourth vaccine doses (Figure 1). Because data collection was still ongoing and there was a demographic difference between the participants who received the third dose of vaccination and those who received the fourth dose of vaccination, subgroup analyses that consider the factors affecting changes in antibody titers should be conducted in a future study. Moreover, we did not measure antibodies to nucleocapsid (N) proteins that indicate a history of infection. Despite these limitations, the findings provide important information regarding the fourth vaccine dose or further boosters, especially for vulnerable groups, such as elderly people. Further research would be needed to determine the appropriate timing of future vaccination.
